# Supplementary figures and images for: Integrative microRNA-gene expression network analysis in genetic hypercalciuric stone-forming rat kidney
Source: PeerJ. 2016 Mar 31;4:e1884. doi: 10.7717/peerj.1884 (PMC4824905; doi:10.7717/peerj.1884)

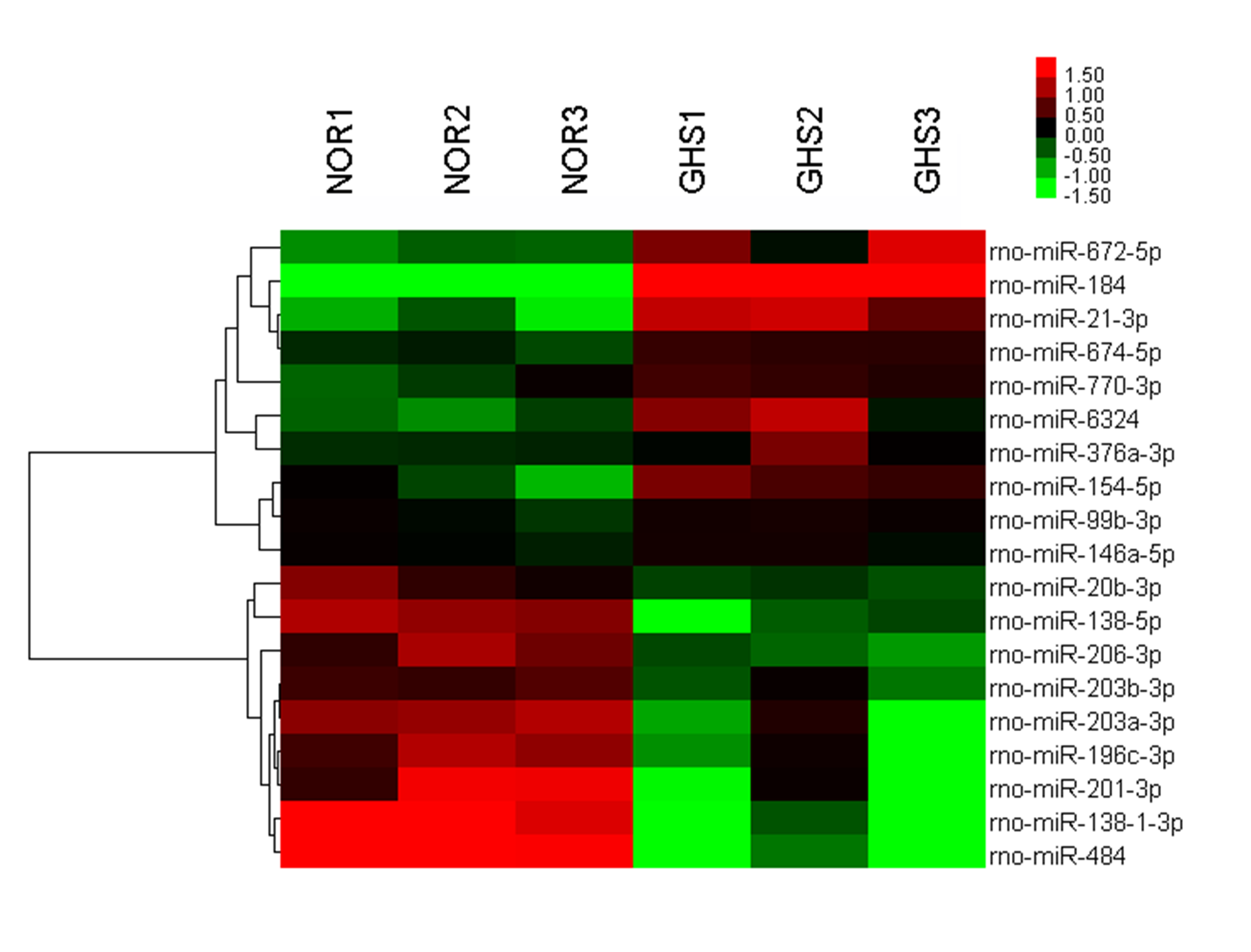

Supplement: Figure S1 — Samples consist of kidneys from three paired rats (GHS and control SD rats). Green, downregulated miRNAs; red, upregulated miRNAs. [file peerj-04-1884-s014.png]
