# Supplementary material for: Integrative microRNA-gene expression network analysis in genetic hypercalciuric stone-forming rat kidney
Source: PeerJ. 2016 Mar 31;4:e1884. doi: 10.7717/peerj.1884 (PMC4824905; doi:10.7717/peerj.1884)
Supplement: File S1 — Detailed principle and methodology of microarray-based analysis. [file peerj-04-1884-s015.docx]

## GO analysis

GO analysis was applied to analyze the main function of the differential expression genes according to the Gene Ontology which is the key functional classification of NCBI, which can organize genes into hierarchical categories and uncover the gene regulatory network on the basis of biological process and molecular function.

Specifically, two-side Fisher’s exact test and $\chi^{2}$test were used to classify the GO category, and the false discovery rate (FDR) was calculated to correct the P-value，the smaller the FDR, the small the error in judging the p-value. The FDR was defined as $FDR=1-\frac{N_{k}}{T}$, where $N_{k}$ refers to the number of Fisher’s test P-values less than$\chi^{2}$test P-values. We computed P-values for the GOs of all the differential genes.

Enrichment provides a measure of the significance of the function: as the enrichment increases, the corresponding function is more specific, which helps us to find those GOs with more concrete function description in the experiment. Within the significant category, the enrichment Re was given by:

$$\mathrm{Re}=(n_{f}/n)/(N_{f}/N)$$

where “$n_{f}$” is the number of flagged genes within the particular category, “$n$” is the total number of genes within the same category, “$N_{f}$” is the number of flagged genes in the entire microarray, and “$N$” is the total number of genes in the microarray.

**References:**

2006. The Gene Ontology (GO) project in 2006. Nucleic Acids Res 34: D322-326.

Ashburner, M., C.A. Ball, J.A. Blake, D. Botstein, H. Butler, J.M. Cherry, A.P. Davis, K. Dolinski, S.S. Dwight, J.T. Eppig, M.A. Harris, D.P. Hill, L. Issel-Tarver, A. Kasarskis, S. Lewis, J.C. Matese, J.E. Richardson, M. Ringwald, G.M. Rubin, and G. Sherlock. 2000. Gene ontology: tool for the unification of biology. The Gene Ontology Consortium. Nat Genet 25: 25-29.

Dupuy, D., N. Bertin, C.A. Hidalgo, K. Venkatesan, D. Tu, D. Lee, J. Rosenberg, N. Svrzikapa, A. Blanc, A. Carnec, A.R. Carvunis, R. Pulak, J. Shingles, J. Reece-Hoyes, R. Hunt-Newbury, R. Viveiros, W.A. Mohler, M. Tasan, F.P. Roth, C. Le Peuch, I.A. Hope, R. Johnsen, D.G. Moerman, A.L. Barabasi, D. Baillie, and M. Vidal. 2007. Genome-scale analysis of in vivo spatiotemporal promoter activity in Caenorhabditis elegans. Nat Biotechnol 25: 663-668.

Schlitt, T., K. Palin, J. Rung, S. Dietmann, M. Lappe, E. Ukkonen, and A. Brazma. 2003. From gene networks to gene function. Genome Res 13: 2568-2576.

## Pathway Analysis

Pathway analysis was used to find out the significant pathway of the differential genes according to KEGG. Still, we turn to the Fisher’s exact test and $\chi^{2}$test to select the significant pathway, and the threshold of significance was defined by P-value and FDR. The enrichment Re was calculated like the equation above.

**References**

Kanehisa, M., S. Goto, S. Kawashima, Y. Okuno, and M. Hattori. 2004. The KEGG resource for deciphering the genome. Nucleic Acids Res 32: D277-280.

Yi, M., J.D. Horton, J.C. Cohen, H.H. Hobbs, and R.M. Stephens. 2006. WholePathwayScope: a comprehensive pathway-based analysis tool for high-throughput data. BMC Bioinformatics 7: 30.

Draghici, S., P. Khatri, A.L. Tarca, K. Amin, A. Done, C. Voichita, C. Georgescu, and R. Romero. 2007. A systems biology approach for pathway level analysis. Genome Res 17: 1537-1545.

## Signal-Net

Gene-gene interaction network was constructed based on the data of differentially expressed genes. Using java that allows users to build and analyze molecular networks, network maps were constructed. For instance, if there is confirmative evidence that two genes interact with each other, an interaction edge is assigned between the two genes. The considered evidence is the source of the interaction database from KEGG. Networks are stored and presented as graphs, where nodes are mainly genes (protein, compound, etc.) and edges represent relation types between the nodes, e.g. activation or phosphorylation. The graph nature of Networks raised our interest to investigate them with powerful tools implemented in R.

To investigate the global network, we computationally identify the most important nodes. To this end we turn to the connectivity (also known as degree) defined as the sum of connection strengths with the other network genes:

$$\text{K}_{i}=\sum_{u\neq i} a_{ui}$$

In gene networks, the connectivity measures how correlated a gene is with all other network genes. For a gene in the network, the number of source genes of a gene is called the indegree of the gene and the number of target genes of a gene is its outdegree. The character of genes is described by betweenness centrality measures reflecting the importance of a node in a graph relative to other nodes. For a graph G:(V,E) with n vertices, the relative betweenness centrality$C'_{B}(v)$ is defined by:

$$C'_{B}(v)=\frac{2}{n^{2}-3n+2}\sum_{\begin{matrix} s\neq v\neq t\in V \\ s\neq t \end{matrix}} \frac{\sigma_{st}(v)}{\sigma_{st}}$$

where$\sigma_{st}$ is the number of shortest paths from s to t, and $\sigma_{st}(v)$is the number of shortest paths from s to t that pass through a vertex v.

**References:**

Jansen, R., D. Greenbaum, and M. Gerstein. 2002. Relating whole-genome expression data with protein-protein interactions. Genome Res 12: 37-46.

Li, C. and H. Li. 2008. Network-constrained regularization and variable selection for analysis of genomic data. Bioinformatics 24: 1175-1182.

Wei, Z. and H. Li. 2007. A Markov random field model for network-based analysis of genomic data. Bioinformatics 23: 1537-1544.

Zhang, J.D. and S. Wiemann. 2009. KEGGgraph: a graph approach to KEGG PATHWAY in R and bioconductor. Bioinformatics 25: 1470-1471.

Spirin, V. and L.A. Mirny. 2003. Protein complexes and functional modules in molecular networks. Proc Natl Acad Sci U S A 100: 12123-12128.

## MicroRNA-gene-network

To build a miRNA-Gene-Network, the relationship between miRNAs and genes was counted by their differential expression values, and according to their interactions in the Sanger miRNA database.

The adjacency matrix of MicroRNA and genes A= [ai,j] was made by the attribute relationships among genes and MicroRNA, where ai,j represents the weight of the relationship between gene i and MicroRNA j. In the miRNA-Gene-Network, the circles represent is represented by one edge. The center of the network was represented by degree. Degree is the contribution of one miRNA to the genes around or the contribution of one gene to the miRNAs around. The key miRNA and gene in the network always have the biggest degrees.

**References:**

Je-Gun, J, Kyu-Baek, H, Jin-Wu, N, Soo-Jin, K, and Byoung-Tak, Z. 2007. Discovery of microRNA-mRNA modules via population-based probabilistic learning. Bioinfo 23:1141.

Reut, S, Daniel, L, Moshe, Oren, Yitzhak, P. 2007. Global and local architecture of the mammalian microRNA-transcription factor regulatory network. PLOS One 3:1291

Anton. E, Bino. J, Ulrike. G, Thomas. T, Chris. S, and Debora. M. 2003. MicroRNA targets in Drosophila. Genome Biol 5:R1.
